# Discovery of a new human T-cell lymphotropic virus (HTLV-3) in Central Africa

**DOI:** 10.1186/1742-4690-2-30

**Published:** 2005-05-09

**Authors:** Sara Calattini, Sébastien Alain Chevalier, Renan Duprez, Sylviane Bassot, Alain Froment, Renaud Mahieux, Antoine Gessain

**Affiliations:** 1 Unité d'Epidémiologie et Physiopathologie des Virus Oncogènes, Institut Pasteur, 28 rue du Dr Roux, 75015 Paris, France; 2 Laboratoire ERMES, IRD, Technoparc, Orléans cedex 2, France

## Abstract

Human T-cell Leukemia virus type 1 (HTLV-1) and type 2 (HTLV-2) are pathogenic retroviruses that infect humans and cause severe hematological and neurological diseases. Both viruses have simian counterparts (STLV-1 and STLV-2). STLV-3 belongs to a third group of lymphotropic viruses which infect numerous African monkeys species. Among 240 Cameroonian plasma tested for the presence of HTLV-1 and/or HTLV-2 antibodies, 48 scored positive by immunofluorescence. Among those, 27 had indeterminate western-blot pattern. PCR amplification of *pol *and *tax *regions, using HTLV-1, -2 and STLV-3 highly conserved primers, demonstrated the presence of a new human retrovirus in one DNA sample. *tax *(180 bp) and *pol *(318 bp) phylogenetic analyses demonstrated the strong relationships between the novel human strain (Pyl43) and STLV-3 isolates from Cameroon. The virus, that we tentatively named HTLV-3, originated from a 62 years old Bakola Pygmy living in a remote settlement in the rain forest of Southern Cameroon. The plasma was reactive on MT2 cells but was negative on C19 cells. The HTLV 2.4 western-blot exhibited a strong reactivity to p19 and a faint one to MTA-1. On the INNO-LIA strip, it reacted faintly with the generic p19 (I/II), but strongly to the generic gp46 (I/II) and to the specific HTLV-2 gp46. The molecular relationships between Pyl43 and STLV-3 are thus not paralleled by the serological results, as most of the STLV-3 infected monkeys have an "HTLV-2 like" WB pattern. In the context of the multiple interspecies transmissions which occurred in the past, and led to the present-day distribution of the PTLV-1, it is thus very tempting to speculate that this newly discovered human retrovirus HTLV-3 might be widespread, at least in the African continent.

## Findings

Three types of Primate T-cell lymphotropic viruses (PTLVs) have been discovered so far in primates [1]. While two of them i.e. PTLV-1 and PTLV-2 include human (HTLV-1, HTLV-2) and simian (STLV-1, STLV-2) viruses, the third type (STLV-3) consists only, so far, of simian strains. Sequence comparisons of STLV-3 proviruses indicated that these strains are highly divergent from HTLV-1 (60% nucleotide similarity), HTLV-2 (62%), or STLV-2 (62%) prototype sequences. In all phylogenetic analyses, STLV-3 viruses cluster in a highly supported group, indicating an evolutionary lineage independent from PTLV-1 and PTLV-2. Nevertheless, STLV-3 lineage is composed of at least three subtypes that are corresponding more or less to the geographical origin of the virus (East, West or Central Africa) [2-9]. Most of the viruses belonging to the PTLV-1 type cannot be separated into distinct phylogenetic lineages according to their species of origin. Their intermixing has therefore been inferred as an evidence for past or recent interspecies transmission episodes. The hypothesis of viral transmission from monkeys to humans is supported by an increasing number of observations [1]. Thus, it has been proposed that HTLV strains related to STLV-3 might infect human populations living in areas where STLV-3 is present.

Cameroon has a remarkable diversity of retroviruses. All the subtypes of HIV-1 group M (A to H) are present, subtype-recombinant strains co-circulate, and HIV-1 groups O and N have been reported. Besides, HTLV-1 subtypes B and D as well as HTLV-2 type A and B are also present in Cameroonian individuals, while STLV-1 and STLV-3 strains have been isolated from several non-human primates (NHPs) species living in this region [3,4,8]. We therefore conducted a study to search for HTLV variants in Cameroonian individuals with HTLV-1/2 indeterminate serology. This survey was approved by both the national (Cameroon Ministry of Health and their National Ethics committee) and local authorities (village chief) with information to each participant. An oral informed consent was obtained from each participant (adults or parents for minors). A series of 240 blood samples was obtained from Bakola (n = 64) and Baka (n = 65) Pygmies, while others (n = 111) were obtained from Bantous (mainly from the Fang, Mvae and Ngumba tribes). All these individuals (117 women and 123 men, mean age 44, range 10–75 years) live in remote villages in the rain forest area of the Southern part of Cameroon.

The 240 plasma were tested at a 1/40 dilution for the presence of HTLV-1/2 antibodies with a highly sensitive immunofluorescence test (IF), that uses MT2 and C19 as HTLV-1 and HTLV-2 viral antigen producing cells respectively. This test also allows the detection of STLV-3 positive samples [4,5]. The 48 plasma that were IF reactive on MT2, C19 or both, were further tested by western blot (WB HTLV BLOT 2.4; Genelabs Diagnostics, Singapore). Among the 48 samples tested, 4 and 11 WB patterns were very evocative of HTLV-1 and HTLV-2 infection respectively, while 27 exhibited diverse HTLV incomplete patterns, including some HTLV-1 indeterminate gag profile (HGIP). Six samples were WB negative. High-molecular weight DNA was extracted from the 48 blood samples and was first subjected to PCR using human β-globin specific primers, to ensure that DNA was amplifiable. They were then subjected to two series of PCR using degenerated *tax *and *pol *primers designed on highly conserved regions that are common to all PTLVs. The *tax *primers are the following: SCTaxoutse: 5'-CTHTAYGGRTACCCHGTCTACGT-3' and SCTaxoutas: 5'-AGGGGAGBCGAGGGATAAGG-3' corresponding to nucleotides 7279 to 7301 and 7455 to 7474 respectively of the prototype STLV-3_PHA969 _sequence (GenBank accession number Y07616). The *pol *primers are SCPOL1outse: 5'-TTAAACCDGARCGCCTCCAGGC-3' (nt 2485 to 2506) SCPOL1outas: 5'-GGDGTDCCYTTRGAGACCCA-3' (nt 3201 to 3220) and SCPOL1inse: 5'-TAYHHAGGRCCAGGMAATAACCC-3' (nt 2556 to 2578).

**Figure 1:**
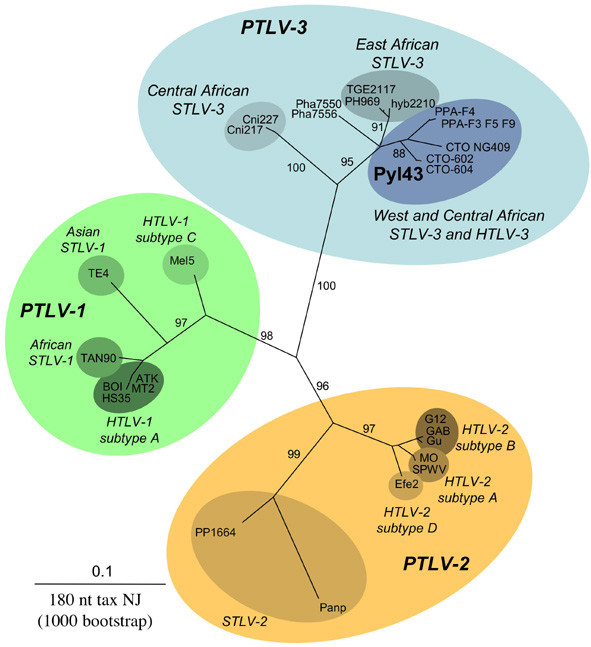
**HTLV-3 is closely related to STLV-3. **Unrooted phylogenetic tree generated with the Neighbor-joining method, performed in the PAUP program (v4.0b10), on a 180 bp fragment of the *tax *gene using all full length PTLV-1/2 available sequences and all published STLV-3 *tax *sequences. The PTLV-1/2/3 strains, including (in bold), the novel sequence generated in this work (Pyl43), were aligned with the DAMBE program (version 4.2.13). The final alignment was submitted to the *Modeltest *program (version 3.6) to select, according to the Akaike Information Criterion (AIC), the best model to apply to phylogenetic analyses. The selected model was the TrN+G. Bootstrap support (1,000 replicates) is noted on the branches of the tree. The branch lengths are drawn to scale, with the bar indicating 0.1 nucleotide replacement per site.

HTLV-1 and HTLV-2 *tax *sequences were obtained for 4 and 11 samples which exhibited complete HTLV-1 and HTLV-2 WB profiles respectively, but none of the WB indeterminate sample gave a PCR signal. Consistent results were obtained for these HTLV-1 and HTLV-2 strains with the *pol *semi-nested PCR. However, a faint band (665 bp) was also obtained for one sample (Pyl43), which was previously found to be *tax *PCR negative. Sequencing of this fragment indicated the presence of an HTLV *pol *sequence that is highly related to STLV-3 strains (86.6% to 99.2% nucleotide identity). Based on an alignment of different STLV-3 sequences, a *tax *semi-nested PCR was then designed using SCTaxoutse (see above) and Mac4 followed by Mac2 and Mac4 as inner primers [10]. This allowed the amplification of a 279 bp fragment which was also found to be highly homologous to STLV-3 strains (92.4% to 99.6% nucleotide identity). We did two phylogenetic analyses (*tax *and *pol*) with the neighbor joining method. Assessment of a 180-bp *tax *sequence (Figure 1) or of a 665-bp *pol *region (data not shown) demonstrated a strong relationship between Pyl43 and STLV-3 strains from Cameroon.

**Figure 2:**
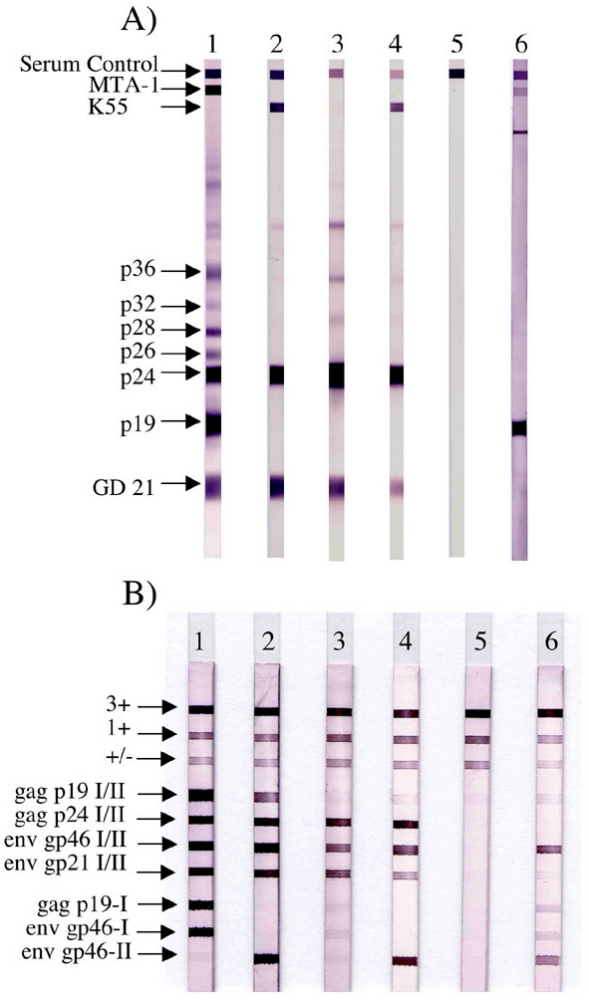
**Serological pattern of the person infected by the HTLV-3 Pyl43 strain**. (A) Western Blot from Genelabs Diagnostics (HTLV BLOT 2.4 version) and (B) a line immunoassay (INNO-LIA HTLV confirmation Immunogenetics). The HTLV 2.4 western blot kit is based on strips incorporating HTLV-1/2 native viral antigens (originating from HTLV-1 infected cells) to which HTLV-1 (MTA-1) or HTLV-2 (K55) gp46s or HTLV-1 and HTLV-2 (GD21) gp21 recombinant proteins have been added [11]. The INNO LIA kit uses only recombinant antigens and synthetic peptides derived from both HTLV-1 and HTLV-2 proteins sequences. Whereas gag p19 I/II corresponds both to a recombinant protein and synthetic peptides being recognized by anti HTLV-1 and HTLV-2 immune sera, env gp46 I/II corresponds only to synthetic peptides recognized by anti HTLV-1 and HTLV-2 immune sera. env gp46 II corresponds to synthetic peptides specific of HTLV-2 [12]. (A, B) Lane 1: HTLV-1 positive control; lane 2: HTLV-2 positive control; lane 3: STLV-3 positive control (STLV-3_604 _strain); lane 4: STLV-3 positive control (STLV-3_F3_); lane 5: HTLV-1/2 negative control; lane 6: plasma from the person infected by HTLV-3 (Pyl43 strain).

The HTLV-3 sample originated from a 62 years old Bakola Pygmy living in a remote settlement in the ocean department of Southern Cameroon. His plasma was reactive on MT2 cells (titer: 1/320) but was negative on C19 cells. The HTLV BLOT 2.4 WB [11] exhibited a strong reactivity to p19 and a faint one to MTA-1 (Figure 2A). On the INNO-LIA strip (Innogenetics, Ghent, Belgium) [12], it reacted faintly (+/-) with the generic p19 (I/II), but strongly to the generic *env *gp46 (I/II) and to the specific HTLV-2 gp46 (Figure 2B). Surprisingly, the close molecular relationship between Pyl43 and STLV-3 is thus not paralleled by the serological results, as most of the STLV-3 infected monkeys have an "HTLV-2 like" WB pattern (p24 > to p19 with or without K55) (Figure 2A, lanes 3–4) [2-9].

In conclusion, we have demonstrated in this report the presence of a new human retrovirus in the peripheral blood cells of a Central African native. This virus is closely related to STLV-3. In the context of multiple interspecies transmissions that have occurred in the past and led to the present-day distribution of the PTLV-1 [1], we suggest that HTLV-3 might be widespread, throughout the African continent. HTLV-3 infection seems to be reflected by an HTLV indeterminate serological WB pattern. This raises an important public health question regarding the effectiveness of the current commercially available screening and confirmation tests for detecting this new HTLV type. Key research priorities are now to investigate the transmission modes of this virus as well as possible pathogenic associations.

## List of abbreviations used

PTLV: Primate T Lymphotropic Viruses

HTLV: Human T Cell Lymphotropic Virus

PCR: Polymerase Chain Reaction

WB: western-blot

NHPs: Non Human Primates

HGIP: HTLV Gag Indeterminate Profile

## Nucleotide accession number

The *tax *and *pol *accession number for the sequences determined in this study are: [GenBank:DQ020492, GenBank:DQ020493] respectively.

## Competing interests

The author(s) declare that they have no competing interests.

## Authors' contributions

SC and SAC performed the laboratory work. SB did the serological assay and RD the phylogenetic analyses. AF and AG organized and performed the field studies, AG and RM designed, implemented and coordinated the study, wrote the manuscript. All authors have read and approved the manuscript.

## Note

Wolfe et al. recently reported in an abstract the presence of two novel HTLV viruses [13]. Whether or not these viruses are related to the new strain described here (HTLV-3 Pyl43) remains to be determined by further comparative studies.
